# Identification of Five Novel* Salmonella* Typhi-Specific Genes as Markers for Diagnosis of Typhoid Fever Using Single-Gene Target PCR Assays

**DOI:** 10.1155/2016/8905675

**Published:** 2016-11-15

**Authors:** Yuan Xin Goay, Kai Ling Chin, Clarissa Ling Ling Tan, Chiann Ying Yeoh, Ja'afar Nuhu Ja'afar, Abdul Rahman Zaidah, Suresh Venkata Chinni, Kia Kien Phua

**Affiliations:** ^1^Institute for Research in Molecular Medicine (INFORMM), Universiti Sains Malaysia (USM), Health Campus, 16150 Kubang Kerian, Kelantan, Malaysia; ^2^Department of Medical Microbiology and Parasitology, Universiti Sains Malaysia (USM), Health Campus, 16150 Kubang Kerian, Kelantan, Malaysia; ^3^Faculty of Applied Sciences, AIMST University, Jalan Bedong-Semeling, 08100 Bedong, Kedah, Malaysia

## Abstract

*Salmonella* Typhi (*S*. Typhi) causes typhoid fever which is a disease characterised by high mortality and morbidity worldwide. In order to curtail the transmission of this highly infectious disease, identification of new markers that can detect the pathogen is needed for development of sensitive and specific diagnostic tests. In this study, genomic comparison of* S*. Typhi with other enteric pathogens was performed, and 6* S*. Typhi genes, that is, STY0201, STY0307, STY0322, STY0326, STY2020, and STY2021, were found to be specific* in silico*. Six PCR assays each targeting a unique gene were developed to test the specificity of these genes* in vitro*. The diagnostic sensitivities and specificities of each assay were determined using 39* S*. Typhi, 62 non-Typhi* Salmonella*, and 10 non-*Salmonella* clinical isolates. The results showed that 5 of these genes, that is, STY0307, STY0322, STY0326, STY2020, and STY2021, demonstrated 100% sensitivity (39/39) and 100% specificity (0/72). The detection limit of the 5 PCR assays was 32 pg for STY0322, 6.4 pg for STY0326, STY2020, and STY2021, and 1.28 pg for STY0307. In conclusion, 5 PCR assays using STY0307, STY0322, STY0326, STY2020, and STY2021 were developed and found to be highly specific at single-gene target resolution for diagnosis of typhoid fever.

## 1. Introduction

To date, there are more than 2,500 serotypes identified within the* Salmonella enterica* species [1]. Most are harmless to humans but one serotype,* Salmonella enterica* subspecies* enterica* serovar Typhi (*S*. Typhi), causes typhoid fever, a severe and life-threatening systemic infection in humans. Worldwide, typhoid fever causes 269,000 deaths from 26.9 million new cases each year [2]. Travellers, children, the elderly, and immune-compromised individuals are especially at risk [3, 4]. The clinical manifestations of typhoid fever are similar to other febrile illnesses. Therefore, diagnosis based on clinical signs and symptoms alone is difficult [5]. The emergence of multidrug-resistant* S*. Typhi strains and development of the typhoid carrier state have further complicated the management of typhoid fever [6, 7]. Delay in diagnosis and initiation of antibiotic treatment can cause serious clinical complications and fatality [8]. Thus, early and correct laboratory diagnosis of typhoid fever is critical to reduce the morbidity and mortality, as well as curtail transmission of the disease.

DNA-based detection methods, such as polymerase chain reaction (PCR), have proven to be sensitive, specific, and rapid compared to conventional culture-based methods for the diagnosis of many infectious diseases [9–11]. Several target genes have been used for* S*. Typhi identification using PCR, such as the O antigen somatic genes (*tyv* and* prt*) [12], H antigen flagellar gene (*fliC-*d) [13], and Vi capsular antigen gene (*viaB*) [14]. However, these genes cannot stand alone as single *S*. Typhi-specific diagnostic marker since they are not specific to* S*. Typhi and are also found in other* Salmonella* serotypes. Thus, these markers provide provisional rather than differential diagnosis of typhoid fever. For example, the* fliC-*d gene of* S*. Typhi shares the same nucleic acid sequence as* S*. Muenchen [15]; the* prt* gene is present in* S*. Typhi,* S*. Paratyphi A, and* S*. Enteritidis [12]; and the* viaB* gene is found not only in* S*. Typhi but also in* S*. Dublin, a few strains of* S*. Paratyphi C [16] and* Citrobacter freundii* [17]. Due to the lack of specificity of these target genes, a combination of different pairs of primers using multiplex PCR [18] or nested PCR [19] are needed to increase the sensitivity and specificity of the PCR diagnostic test. This, however, will increase the cost, time, and complexity of the laboratory diagnosis.

Diagnostic markers which can detect pathogens at single-gene target resolution could lead to a simpler, cost-effective, and more functional DNA-based detection method since less primers are needed for target detection. Many approaches, such as subtractive hybridization [20], next generation sequencing [21], and microarray [22] techniques, have been used to identify genes that are specific or unique to a pathogen. However, these high-end technologies are cumbersome and expensive and sometimes yield false negative or false positive results [23]. Since bacterial genome databases have expanded tremendously over the past decade and advancement in computing technologies has made nucleic acid sequence alignment services readily accessible at NCBI,* in silico* comparative hybridization approach coupled with* in vitro* PCR (wet-lab) validation is sufficient to facilitate the translation of genomic data into diagnostic marker discoveries. In this study, a low-cost and simple attempt was made to identify new DNA diagnostic markers specific for* S*. Typhi by utilizing genome data (stored in NCBI databases) and nucleic acid sequence alignment tools (BLASTn) that are readily available in the public domain. The diagnostic sensitivities and specificities of the primers designed for amplifying whole gene sequences can be validated using a panel of confirmed bacteria isolates selected from* S*. Typhi, non-Typhi* Salmonella*, and non-*Salmonella* clinical isolates. To serve as a control for the PCR reaction, 16S rRNA gene, that is ubiquitous among bacteria species, can be used as a PCR amplification control [24].

## 2. Materials and Methods

### 2.1. Bacterial Strains

A total of 111 bacteria isolates including 39* S*. Typhi, 62 non-Typhi* Salmonella* serotypes, and 10 non-*Salmonella* strains were used in this study.* S*. Typhi strains consisted of 1* S*. Typhi reference strains (ATCC 7251) and 38 different pulsed-field types (PFTs) representing all strains in the state of Kelantan in Malaysia. These 38 PFTs were the result of screening 279* S*. Typhi clinical isolates using pulsed-field gel electrophoresis (PFGE) [25]. Non-Typhi* Salmonella* serotypes were closely related* Salmonella* species made up of 26 different serotypes (Table 2) and 10 ATCC strains including* S*. Paratyphi A (ATCC 9150),* S*. Paratyphi B (ATCC BAA 1250),* S*. Paratyphi C (ATCC 9068),* S*. Enteritidis (ATCC 13076),* S*. Typhimurium (ATCC 14028),* S*. Weltevreden (NCTC 6534),* S*. Agona (ATCC 51957),* S*. Heidelberg (ATCC 8326),* S*. Poona (ATCC 04840), and* S*. Braenderup (ATCC BAA-664). In addition, 10 other non-*Salmonella* strains such as* Shigella dysenteriae*,* Shigella flexneri*,* Shigella boydii*,* Shigella sonnei*,* Vibrio cholera, Enterohemorrhagic E*.* coli*,* Enteropathogenic E*.* coli*,* Aeromonas hydrophila*,* Yersinia enterocolitica,* and* Klebsiella pneumonia* were also included. All clinical strains were procured from the Department of Clinical Microbiology and Parasitology, Hospital Universiti Sains Malaysia (HUSM), Kelantan, Malaysia, and the Biobank of the Institute for Research in Molecular Medicine (INFORMM), Kelantan, Malaysia. All bacteria strains were stored in glycerol stocks at −80°C until being ready for use. Ethical clearance for this project was obtained from the Human Research Ethics Committee, Universiti Sains Malaysia (reference number USMKK/PPP/JEPeM [235.3.(16)]).

### 2.2. Culture Conditions and Confirmation Tests

All bacteria isolates used in this study were confirmed by traditional culture, biochemical, and serotyping methods as described in ISO6579 with some modifications. Bacteria isolates were revived from frozen glycerol stocks by pipetting 100 *μ*L thawed cells into 10 mL nutrient broth and incubated at 37°C for 18 hours in an orbital shaker at 200 rpm. The bacteria were streaked on Xylose Lysine Deoxycholate (XLD) selective agar and incubated at 37°C for 18 hours. Colonies grown on the agar were tested with a panel of biochemical tests, including Triple Sugar Iron (TSI), urease, Methyl Red Voges Proskauer (MRVP), citrate, and indole tests. Suspected* Salmonella* isolates were then sent to the* Salmonella* Reference Centre, Institute for Medical Research (IMR), Malaysia, to confirm their serotypes using specific antisera and latex agglutination method.

### 2.3. Identification of* S*. Typhi-Specific Genes Using Bioinformatics (*In Silico*)

**Figure 1:**
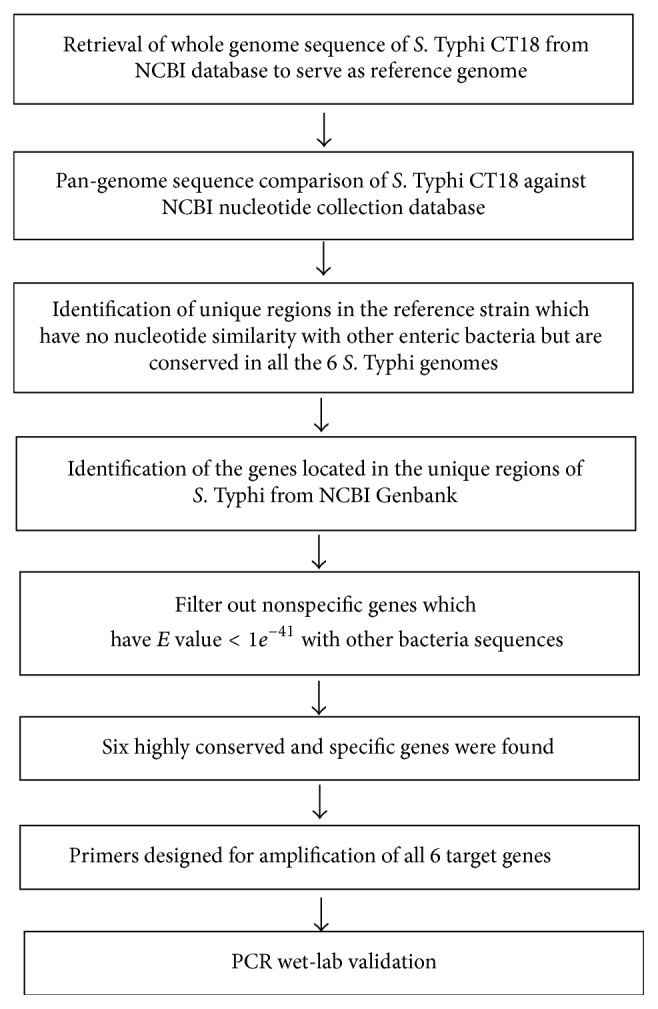
Experimental workflow describing the comparative genomic and wet-lab approaches used to identify and validate* S*. Typhi-specific DNA diagnostic markers.

Full genome sequence of *S*. Typhi CT18 (GenBank accession number AL513382) was downloaded from the National Center for Biotechnology Information database (NCBI) and used as the reference genome. The 2 plasmids, namely, pHCM1 and pHCM2, which resided in *S*. Typhi CT18 were excluded since plasmids are genetically unstable. The 6 complete *S*. Typhi whole-genome sequences available in NCBI were used for data mining. They comprised CT18 (Genbank accession number AL513382) [27], Ty2 (Genbank accession number AE014613) [28], P-stx-12 (Genbank accession number CP003278) [29], Ty21a (Genbank accession number CP002099) [30], B/SF/13/03/195 (Genbank accession number CP012151) [31], and PM016/13 (Genbank accession number CP012091) [32]. In order to ascertain whether the genomic regions were conserved and specific to *S*. Typhi, the nucleotide Basic Local Alignment Search Tool (BLASTn), a free online software for nucleic acid analysis, was used to compare the whole-genome sequence of *S*. Typhi CT18 with the other 5 complete *S*. Typhi genomes and other bacteria genomes in the NCBI database (https://blast.ncbi.nlm.nih.gov/Blast.cgi). Genes found in unique regions which have no nucleotide similarity with other enteric organisms were identified and retrieved from the Genebank of NCBI. These genes were further screened individually using similarity searches against the NCBI non-redundant nucleotide (nr/nt) database to reconfirm their specificities. The program was set for “somewhat similar sequences search,” which allowed nucleotide sequence matching down to 7 bases (the smaller the nucleotide size, the more sensitive the result). Realizing the high genome similarity among the enteric pathogens and the possibility that different geographical areas may result in different bacterial genotypes, only genes which have 100% sequence conservation (an *E*-value threshold = 0.0) in all 6 complete *S*. Typhi genomes and had little or no similarity (*E*-value threshold ≥ 1*e* ^−41^) to other bacterial sequences in the NCBI database were considered as potential targets and were subjected to wet-lab analysis. The experimental pipeline is as shown in Figure 1.

### 2.4. Design of Oligonucleotide Primers for PCR Amplification

Primers were designed manually to amplify the *S*. Typhi-specific genes identified previously, including the start and the stop codons. A pair of primers specific for 16S rRNA gene amplification as described by Marchesi and colleagues [24] were also incorporated into each PCR assay to serve as an internal amplification control (IAC). This is a universal gene target which is highly conserved in bacteria [24]. All primers were synthesized by Integrated DNA Technologies (IDT) Pte. Ltd., Malaysia.

### 2.5. Template DNA Extraction

DNA from all bacteria isolates were extracted using DNeasy Blood & Tissue kit® (Qiagen, USA) according to the manufacturer's instructions. The purity and concentration of the extracted DNA were determined using Nanodrop Spectrophotometer ND-1000 (Thermo Fisher Scientific, USA). DNA concentration was measured from the absorbance at 260 nm. Ratio of the absorbance at 260 and 280 nm (*A*_260/280_) and ratio of the absorbance at 230 and 260 nm (*A*_230/260_) were used to evaluate the DNA quality. The extracted DNAs were diluted to a final stock concentration of 50 ng/*μ*L using ultrapure water and stored at −20°C until ready for PCR amplification.

### 2.6. Optimization of PCR

Each PCR assay was optimized using a modified Taguchi method as described by Cobb and Clarkson [33]. The effects and interactions of the 4 main PCR components (IAC primers,* S*. Typhi-specific gene primers, MgCl_2_, and annealing temperatures) each at 3 different levels (IAC primers: 0.05, 0.10, and 0.15 *μ*M;* S*. Typhi primers: 1.00, 1.50, and 2.00 *μ*M; MgCl_2_: 2.00, 2.50, and 3.00 mM, and annealing temperatures: 50, 55, and 60°C) were investigated in a balanced orthogonal array of 9 experimental combinations. The PCR amplifications were carried out in a total reaction volume of 20 *μ*L, and the PCR products were analysed on a 1.2% (w/v) agarose gel containing SYBR® Safe DNA Gel Stain (Invitrogen, USA), visualized using a blue-light transilluminator (Syngene, UK).

### 2.7. Analytical Specificities of Genes Unique to* S*. Typhi

Analytical specificities of the PCR assays were assessed by running each PCR assay on a panel of bacteria strains consisting of 39* S*. Typhi, 62 non-Typhi* Salmonella*, and 10 non-*Salmonella* clinical isolates.

### 2.8. Detection Limit of the PCR Assays

Detection limit of the PCR assays was defined as the minimum amount of *S*. Typhi DNA (ng/*μ*L) that yielded positive PCR amplicons. The assay sensitivities were determined by amplification of a 5-fold serial dilution of *S*. Typhi ATCC 7251 DNA, ranging from 50 ng to 25.6 fg. Two microliters of the DNA was subjected to PCR amplification. The analytical sensitivity was indicated by the presence of visible PCR product bands on the agarose gel using the transilluminator as described above.

### 2.9. DNA Sequencing

To confirm the PCR products were indeed derived from the *S*. Typhi strains, PCR amplicons from all assays produced using Phusion® High-Fidelity DNA Polymerase (New England Biolabs, USA) were purified and sent to First BASE Laboratories Pte. Ltd., Malaysia, for sequencing. The resultant nucleotide sequences were compared with the reference *S*. Typhi CT18 gene sequences in NCBI using BioEdit software.

## 3. Results

Using the bioinformatic method for whole-genome comparison (Figure 1), 6 potential diagnostic markers with NCBI locus tags, STY0201, STY0307, STY0322, STY0326, STY2020, and STY2021, were found. They exhibit 100% query coverage and identity (*E*-value = 0) with all 6* S*. Typhi gene sequences but had low or no significant similarity (*E*-value ≥ 1*e* ^−41^) with other enteric bacteria nucleotide sequences as of 11 March 2016. These genes were found to be (bioinformatically) highly conserved and specific and thus were selected for further wet-lab validation using PCR method. The primers designed to amplify these selected genes are shown in Table 1.

The results showed that all 6 designed primer pairs successfully amplified their target genes with amplicon sizes of 1176, 495, 678, 261, 429, and 732 bps, respectively. DNA sequencing results of the amplicons showed 100% identity with their corresponding *S*. Typhi genes, confirming the fidelity and sensitivity of the primers.

The 6 single-gene target PCR assays were then optimized using Taguchi method with the incorporation of IAC which targeted the 16S rRNA gene. The optimized master mix for the PCR assays targeting STY0201, STY0307, and STY2020 genes consisted of 1x Green GoTaq Flexi Buffer, 2.0 mM MgCl_2_, 0.2 mM dNTPs, 1.5 *μ*M *S*. Typhi-specific gene primers, 0.10 *μ*M IAC primers, 0.75 U GoTaq Flexi DNA Polymerase (Promega, USA), and 5% glycerol in a total volume of 20 *μ*L. Two microliters of test DNA (50 ng/*μ*L) was added to the master mix and amplified using the following optimized thermal-cycling parameters: initial denaturation at 95°C for 1 min, followed by 30 cycles elongation at 95°C for 30 s, 55°C for 30 s, 72°C for 1 min and a final extension at 72°C for 5 min. Similar PCR conditions were used for amplification of STY0322, STY0326, and STY2021 genes except for the concentration of MgCl_2_ and IAC primers which were set at 3.0 mM and 0.15 *μ*M, respectively. The optimal annealing temperature was set at 50°C. Under these conditions, the IAC primer pair produced an amplicon of 1,362 bp for all bacteria isolates tested (111/111).

**Figure 2:**
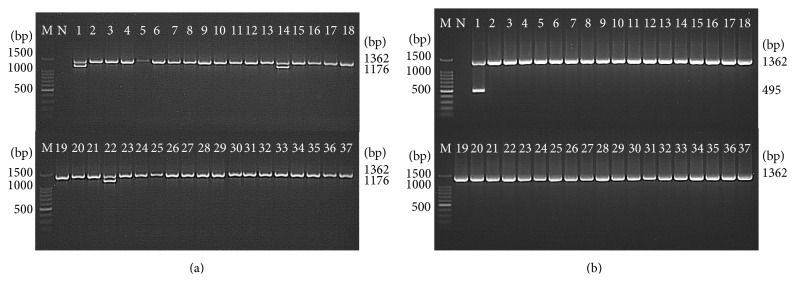
Analytical specificity of the PCR assay for detection of (a) STY0201 and (b) STY0307 genes (representative figures). Lane: M = 100 bp DNA ladder (Promega); N = negative control; 1 =* S*. Typhi; 2 =* S*. Paratyphi A; 3 =* S*. Paratyphi B; 4 =* S*. Paratyphi C; 5 =* S*. Enteritidis; 6 =* S*. Typhimurium; 7 =* S*. Weltevreden; 8 =* S*. Agona; 9 =* S*. Heidelberg; 10 =* S*. Poona; 11 =* S*. Hadar; 12 =* S*. Braenderup; 13 =* S*. Albany; 14 =* S*. Oslo; 15 =* S*. Kibi; 16 =* S*. Newport; 17 =* S*. Tshiongwe; 18 =* S*. Uppsala; 19 =* S*. Richmond; 20 =* S*. Bardo; 21 =* S*. Emek; 22 =* S*. Kissi; 23 =* S*. Virchow; 24 =* S*. Bordeaux; 25 =* S*. Regent; 26 =* S*. Java; 27 =* S*. Farsta; 28 =* Shigella dysenteriae*; 29 =* Shigella flexneri*; 30 =* Shigella sonnei*; 31 =* Shigella boydii*; 32 =* Vibrio cholerae*; 33 =* Enterohemorrhagic Escherichia coli*; 34 =* Enteropathogenic Escherichia coli*; 35 =* Aeromonas hydrophila*; 36 =* Yersinia enterocolitica*; and 37 =* Klebsiella pneumonia*. The PCR amplicon sizes for genes 16S rRNA, STY0201, and STY0307 were 1326 bp, 1176 bp, and 732 bp, respectively.

**Figure 3:**
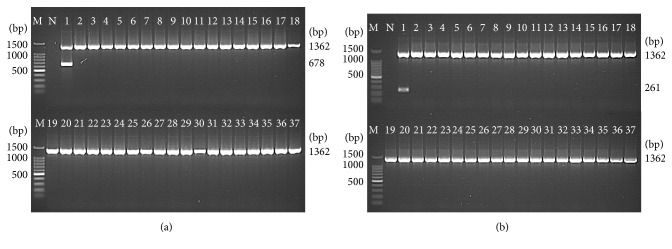
Analytical specificity of the PCR assay for detection of (a) STY0322 and (b) STY0326 genes, respectively (representative gels). Lane: M = 100 bp DNA ladder (Promega); N = negative control; 1 =* S*. Typhi; 2 =* S*. Paratyphi A; 3 =* S*. Paratyphi B; 4 =* S*. Paratyphi C; 5 =* S*. Enteritidis; 6 =* S*. Typhimurium; 7 =* S*. Weltevreden; 8 =* S*. Agona; 9 =* S*. Heidelberg; 10 =* S*. Poona; 11 =* S*. Hadar; 12 =* S*. Braenderup; 13 =* S*. Albany; 14 =* S*. Oslo; 15 =* S*. Kibi; 16 =* S*. Newport; 17 =* S*. Tshiongwe; 18 =* S*. Uppsala; 19 =* S*. Richmond; 20 =* S*. Bardo; 21 =* S*. Emek; 22 =* S*. Kissi; 23 =* S*. Virchow; 24 =* S*. Bordeaux; 25 =* S*. Regent; 26 =* S*. Java; 27 =* S*. Farsta; 28 =* Shigella dysenteriae*; 29 =* Shigella flexneri*; 30 =* Shigella sonnei*; 31 =* Shigella boydii*; 32 =* Vibrio cholerae*; 33 =* Enterohemorrhagic Escherichia coli*; 34 =* Enteropathogenic Escherichia coli*; 35 =* Aeromonas hydrophila*; 36 =* Yersinia enterocolitica*; and 37 =* Klebsiella pneumonia*. The PCR amplicon sizes for genes 16S rRNA, STY0322, and STY0326 were 1,326 bp, 678 bp, and 261 bp, respectively.

**Figure 4:**
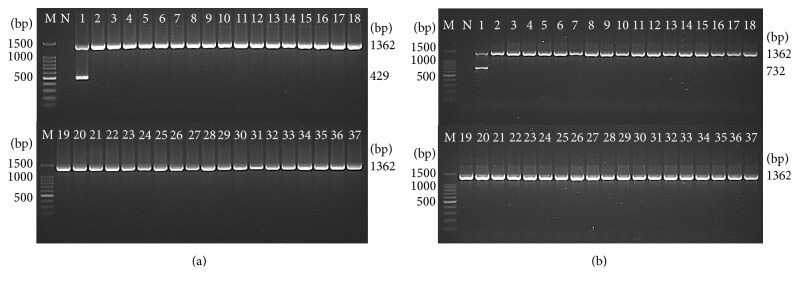
Analytical specificity of the PCR assay for detection of (a) STY2020 and (b) STY2021 genes, respectively (representative figures). Lane: M = 100 bp DNA ladder (Promega); N = negative control; 1 =* S*. Typhi; 2 =* S*. Paratyphi A; 3 =* S*. Paratyphi B; 4 =* S*. Paratyphi C; 5 =* S*. Enteritidis; 6 =* S*. Typhimurium; 7 =* S*. Weltevreden; 8 =* S*. Agona; 9 =* S*. Heidelberg; 10 =* S*. Poona; 11 =* S*. Hadar; 12 =* S*. Braenderup; 13 =* S*. Albany; 14 =* S*. Oslo; 15 =* S*. Kibi; 16 =* S*. Newport; 17 =* S*. Tshiongwe; 18 =* S*. Uppsala; 19 =* S*. Richmond; 20 =* S*. Bardo; 21 =* S*. Emek; 22 =* S*. Kissi; 23 =* S*. Virchow; 24 =* S*. Bordeaux; 25 =* S*. Regent; 26 =* S*. Java; 27 =* S*. Farsta; 28 =* Shigella dysenteriae*; 29 =* Shigella flexneri*; 30 =* Shigella sonnei*; 31 =* Shigella boydii*; 32 =* Vibrio cholerae*; 33 =* Enterohemorrhagic Escherichia coli*; 34 =* Enteropathogenic Escherichia coli*; 35 =* Aeromonas hydrophila*; 36 =* Yersinia enterocolitica*; and 37 =* Klebsiella pneumonia*. The PCR amplicon sizes for genes 16S rRNA, STY2020, and STY2021 were 1,326 bp, 429 bp, and 732 bp, respectively.

The optimized PCR assays for STY0307, STY0322, STY0326, STY2020, and STY2021 correctly identified all *S*. Typhi (39/39) isolates, whereas none of the non-Typhi* Salmonella* (0/62) and none of the non-*Salmonella* (0/10) isolates were detected. This showed a 100% sensitivity and 100% specificity for the PCR assays (Table 2) and indicate that the 5 genes were unique to *S*. Typhi (Figures 2, 3, and 4).

The results of serial dilution of *S*. Typhi genomic DNA showed that the detection limit of the optimized PCR assays was 32 pg for gene STY0322, 6.4 pg for genes STY0326, STY2020, and STY2021, and 1.28 pg for gene STY0307.

Although gene STY0201 exhibited 100% sensitivity (detection of 39/39* S.* Typhi isolates), it showed cross-reactivity with *S*. Oslo and *S*. Kissi (Table 2), resulting in a specificity of only 97.2% (detection of 2/72 of non-Typhi isolates). Sequencing of their PCR products showed a substitution of nucleotide C → T at position 89 and T → C at positions 354 and 1,026 for both *S*. Kissi and *S*. Oslo. The sequence variation between *S*. Kissi and *S*. Oslo with the *S*. Typhi CT18 reference genome was very small (only 3 nucleotide differences), indicating that the false positive results were due to sequence similarity among themselves.

## 4. Discussion

The diagnosis of typhoid fever based on clinical signs and symptoms is often ambiguous, while phenotypic detection of *S*. Typhi bacteria based on biochemical and serotyping methods is laborious and time-consuming. Thus, rapid molecular detection methods, such as nucleic acid-based amplification, such as PCR assay, is critically needed to help diagnose this contagious disease. Development of this test requires diagnostic markers that are sensitive and specific.

This is the first report on the use of genes STY0307, STY0322, STY0326, STY2020, and STY2021 as *S*. Typhi-specific diagnostic markers. Unlike other *S*. Typhi PCR targets that were selected based on immunological properties, these genes are individually highly specific for *S*. Typhi and therefore can be used as single-gene target PCR assays without the need for nested or multiplex PCR. Also, these targets are whole gene sequences (from start to stop codon for the purpose of whole gene amplification) unlike other diagnostic markers which are only partial gene sequences. The idea of using this strategy is that if the whole gene sequence is specific to the bacteria then primers can be designed at any location of the gene. Thus, these gene sequences not only serve as specific targets for PCR assay, but also are suitable for more advance diagnostic tests that require multiple DNA sites, such as loop-mediated isothermal amplification (LAMP) and strand displacement amplification (SDA) which requires multiple primer annealing sites [34]. These genes could be utilized for the development of innovative Point-of-Care (POC) diagnostics to address the need for low-cost, simple, rapid, and accurate diagnostics for low resource settings.

The gene STY0201 has been used as a PCR target, and the PCR assays that were developed based on this gene were reported to be 100% sensitivity and specificity [35, 36]. However, this study found that this gene was only 97.2% specific and cross-reacted with *S*. Oslo and *S*. Kissi. The incorrect bioinformatic prediction of the specificity of gene STY0201 may be due to the incomplete genome sequence available for the 2 bacteria in the NCBI database that limit the matching accuracy of the BLASTn search. This is a limitation of the alignment-based marker identification method, as it relies on the availability of a complete genome sequence. Thus, whenever new sequence data becomes available for the target organism, the bioinformatic analysis should be repeated to align the current diagnostic markers with the new gene sequence to ensure the specificity.

The other 5 genes identified in this study showed no sequence homology to proteins of known function using protein BLAST (BLASTp) programs. Genes STY0307, STY0322, and STY0326 encode for hypothetical proteins, while genes STY2020 and STY2021 encode for putative bacteriophage proteins. Interestingly, genes STY0307, STY0322, and STY0326 are located in the* Salmonella* Pathogenicity Island 6 (SPI-6). Yet, their role in bacteria virulence and pathogenicity remains unknown. More importantly, antigenicity prediction scores using SCRATCH protein prediction software [26] showed that genes STY0201, STY0207, STY0307, STY0326, and STY2020 were highly antigenic and may have potential to serve as antigens for serodiagnosis of typhoid fever (Table 3). When compared with the deduced amino acid sequence of *S*. Paratyphi A, which is the closest relative of *S*. Typhi [37], the putative proteins showed weak or no similarity to *S*. Typhi (Table 3). These findings provide an opportunity for gene cloning and protein expression to investigate their serodiagnostic value for development of low-cost antibody-based diagnostic tests or vaccines for typhoid fever.

In conclusion, 5* S.* Typhi-specific genes, namely, STY0307, STY0322, STY0326, STY2020, and STY2021, were found to be highly conserved among *S*. Typhi strains. Wet-lab experiments found no false positive reaction with non-Typhi serotypes or non-*Salmonella* enteric pathogens. These genes could serve as useful diagnostic markers for development of DNA-based diagnostics for sensitive and specific detection of typhoid fever.

## Figures and Tables

**Table 1:** List of primers targeting *S.* Typhi-specific genes for the development of 6 PCR assays.

Target genes	Primer labels	Primer sequences (5′-3′)	Target lengths (bp)
STY0201	0201F	ATGCTTTTAAAAAACACAACATGG	1176
	0201R	TTACGGATAGGTGATTGAAAATTG	
STY0307	0307F	ATGAAACCTTTATTCTCAGTGC	495
	0307R	TTAGCGTAATTCCCAGAACC	
STY0322	0322F	ATGAAATATAAAAAAATAAGAG	678
	0322R	CTATGGATTCATTTCCATTTC	
STY0326	0326F	ATGAATACGAATAATTCACC	261
	0326R	TTACCCTCCCCATGTCAC	
STY2020	2020F	ATGCCTGTTATGCATAATTG	429
	2020R	TTATGCTGTTAACGAGTCGTC	
STY2021	2021F	ATGAGTTTAGCGCAGCCTAAATCC	732
	2021R	TTAGAAGTCTCCTGCCTGGAAAC	
16S rRNA^a^	16SF	CAGGCCTAACACATGCAAGTC	1362
	16SR	GGGCGGTGTGTACAAGGC	

^a^16S ribosomal RNA gene served as internal amplification control (IAC) [24].

F represents forward primer.

R represents reverse primer.

**Table 2:** Evaluation of the specificities of the 6 target genes for identification of *S.* Typhi using PCR (total of 111 clinical isolates).

Test bacteria strains	Positive PCR amplification for each target gene					
	STY0201	STY0307	STY0322	STY0326	STY2020	STY2021
*S.* Typhi (*n* = 39)	39/39	39/39	39/39	39/39	39/39	39/39
*S*. Paratyphi A (*n* = 10)	0/10	0/10	0/10	0/10	0/10	0/10
*S*. Paratyphi B (*n* = 10)	0/10	0/10	0/10	0/10	0/10	0/10
*S.* Paratyphi C (*n* = 1)	0/1	0/1	0/1	0/1	0/1	0/1
*S.* Enteritidis (*n* = 10)	0/10	0/10	0/10	0/10	0/10	0/10
*S.* Typhimurium (*n* = 10)	0/10	0/10	0/10	0/10	0/10	0/10
*S*. Weltevreden (*n* = 1)	0/1	0/1	0/1	0/1	0/1	0/1
*S. *Agona (*n* = 1)	0/1	0/1	0/1	0/1	0/1	0/1
*S*. Hadar (*n* = 1)	0/1	0/1	0/1	0/1	0/1	0/1
*S*. Heidelberg (*n* = 1)	0/1	0/1	0/1	0/1	0/1	0/1
*S*. Poona (*n* = 1)	0/1	0/1	0/1	0/1	0/1	0/1
*S*. Braenderup (*n* = 1)	0/1	0/1	0/1	0/1	0/1	0/1
*S.* Albany (*n* = 1)	0/1	0/1	0/1	0/1	0/1	0/1
*S. *Oslo (*n* = 1)	1/1	0/1	0/1	0/1	0/1	0/1
*S*. Kibi (*n* = 1)	0/1	0/1	0/1	0/1	0/1	0/1
*S*. Newport (*n* = 1)	0/1	0/1	0/1	0/1	0/1	0/1
*S.* Tshiongwe (*n* = 1)	0/1	0/1	0/1	0/1	0/1	0/1
*S*. Uppsala (*n* = 1)	0/1	0/1	0/1	0/1	0/1	0/1
*S*. Richmond (*n* = 1)	0/1	0/1	0/1	0/1	0/1	0/1
*S*. Bardo (*n* = 1)	0/1	0/1	0/1	0/1	0/1	0/1
*S*. Emek (*n* = 1)	0/1	0/1	0/1	0/1	0/1	0/1
*S*. Kissi (*n* = 1)	1/1	0/1	0/1	0/1	0/1	0/1
*S*. Virchow (*n* = 1)	0/1	0/1	0/1	0/1	0/1	0/1
*S*. Bordeaux (*n* = 1)	0/1	0/1	0/1	0/1	0/1	0/1
*S*. Regent (*n* = 1)	0/1	0/1	0/1	0/1	0/1	0/1
*S.* Java (*n* = 1)	0/1	0/1	0/1	0/1	0/1	0/1
*S.* Farsta (*n* = 1)	0/1	0/1	0/1	0/1	0/1	0/1
*Shigella dysenteriae* (*n* = 1)	0/1	0/1	0/1	0/1	0/1	0/1
*Shigella flexneri* (*n* = 1)	0/1	0/1	0/1	0/1	0/1	0/1
*Shigella sonnei *(*n* = 1)	0/1	0/1	0/1	0/1	0/1	0/1
*Shigella boydii* (*n* = 1)	0/1	0/1	0/1	0/1	0/1	0/1
*Vibrio cholerae *(*n* = 1)	0/1	0/1	0/1	0/1	0/1	0/1
*Enterohemorrhagic E. coli* (*n* = 1)	0/1	0/1	0/1	0/1	0/1	0/1
*Enteropathogenic E. coli* (*n* = 1)	0/1	0/1	0/1	0/1	0/1	0/1
*Aeromonas hydrophila *(*n* = 1)	0/1	0/1	0/1	0/1	0/1	0/1
*Yersinia enterocolitica* (*n* = 1)	0/1	0/1	0/1	0/1	0/1	0/1
*Klebsiella pneumoniae* (*n* = 1)	0/1	0/1	0/1	0/1	0/1	0/1

**Table 3:** Details of the 5 target genes and their description, antigenicity prediction, protein coverage, and identity with *S. *Paratyphi A.

Number	Target genes (NCBI locus tag)	Gene description	GC content (%)	Antigenicity prediction^*∗*^	Protein coverage with *S. *Paratyphi A (%)	Protein identity with *S. *Paratyphi A (%)
1	STY0307	Hypothetical protein	43	0.66	0	0
2	STY0322	Hypothetical protein	29	0.37	21	33
3	STY0326	Conserved hypothetical protein	37	0.79	0	0
4	STY2020	Putative bacteriophage protein	42	0.66	0	0
5	STY2021	Putative bacteriophage protein	42	0.27	0	0

^*∗*^Antigenicity of the *S. *Typhiproteins predicted using SCRATCH Protein Prediction software [26].
